# YOLO-BCD: A Lightweight Multi-Module Fusion Network for Real-Time Sheep Pose Estimation

**DOI:** 10.3390/s25092687

**Published:** 2025-04-24

**Authors:** Chaojie Sun, Junguo Hu, Qingyue Wang, Chao Zhu, Lei Chen, Chunmei Shi

**Affiliations:** 1 College of Mathematics and Computer Science, Zhejiang A&F University, Hangzhou 311300, China; 2023111021006@stu.zafu.edu.cn (C.S.);; 2 Zhejiang Provincial Key Laboratory of Forestry Intelligent Monitoring and Information Technology Research, Zhejiang A&F University, Hangzhou 311300, China; 3 College of Chemistry and Materials Engineering, Zhejiang A&F University, Hangzhou 311300, China

**Keywords:** sheep posture estimation, graph convolutional network, deep learning

## Abstract

The real-time monitoring of animal postures through computer vision techniques has become essential for modern precision livestock management. To overcome the limitations of current behavioral analysis systems in balancing computational efficiency and detection accuracy, this study develops an optimized deep learning framework named YOLOv8-BCD specifically designed for ovine posture recognition. The proposed architecture employs a multi-level lightweight design incorporating enhanced feature fusion mechanisms and spatial-channel attention modules, effectively improving detection performance in complex farm environments with occlusions and variable lighting. Our methodology introduces three technical innovations: (1) Adaptive multi-scale feature aggregation through bidirectional cross-layer connections. (2) Context-aware attention weighting for critical region emphasis. (3) Streamlined detection head optimization for resource-constrained devices. The experimental dataset comprises 1476 annotated images capturing three characteristic postures (standing, lying, and side lying) under practical farming conditions. Comparative evaluations demonstrate significant improvements over baseline models, achieving 91.7% recognition accuracy with 389 FPS processing speed while maintaining 19.2% parameter reduction and 32.1% lower computational load compared to standard YOLOv8. This efficient solution provides technical support for automated health monitoring in intensive livestock production systems, showing practical potential for large-scale agricultural applications requiring real-time behavioral analysis.

## 1. Introduction

In recent years, with the increasing demand for sheep health management in animal husbandry, the external behavior of sheep, which serves as a comprehensive reflection of their health status, has gained significant attention. The activity level of sheep can be expressed through daily postures, such as standing, lying, and side lying, as sheep typically exhibit reduced activity and an increase in lying or side-lying behaviors when ill. To minimize economic losses, effective monitoring and timely identification of abnormal behaviors are crucial [1,2]. Research indicates that changes in livestock behavior are direct responses to changes in the surrounding environment [3]. Therefore, accurately identifying individual sheep postures plays a key role in the management of sheep farms. Traditional sheep posture recognition mainly relies on manual observation and recording or the use of various sensors [4,5,6]. However, the use of sensors not only may induce stress responses in animals but also has drawbacks such as high costs, high time consumption, and subjectivity. In recent years, computer vision technology has gradually become a hotspot in the field of animal behavior monitoring due to its widespread application in object detection and recognition. The application of deep learning technology, in particular, has been significant, and non-contact detection has been widely promoted in animal husbandry [7,8,9,10]. Some researchers have begun to use deep convolutional neural networks (DCNN) to analyze sheep’s movement behavior in order to monitor their health status and behavioral patterns [11,12,13,14,15]. For instance, studies have shown that using multi-view image fusion techniques can significantly improve the accuracy of sheep posture recognition [16,17,18]. This multi-view fusion method, which combines image information from different angles, not only enhances the robustness of posture estimation but also improves adaptability in complex backgrounds [19]. Furthermore, the development of real-time video analysis technology has enabled researchers to track the dynamic behaviors of sheep more efficiently, thereby optimizing management [20,21,22]. Based on this, using deep learning models to detect sheep behavior and posture can enable real-time monitoring of the sheep’s activity range, drinking, and feeding behaviors, providing important support for the refined management of animal husbandry. Despite certain progress in sheep posture estimation research, several challenges remain. The dynamic characteristics of sheep herds and their movement patterns in confined spaces reduce the accuracy of posture recognition in crowded or highly disturbed environments. Therefore, future research needs to explore more advanced algorithms to enhance recognition accuracy in complex dynamic settings [23,24,25,26,27]. Additionally, the differences in posture estimation across different sheep breeds and growth stages should be further addressed, as this will help develop more personalized monitoring and management solutions. In practical applications, deep learning technology offers a wide range of scenarios for sheep behavior recognition, including health monitoring, breeding management, and feed optimization. Automated posture estimation can significantly improve farming efficiency and economic benefits. In-depth research on sheep posture estimation not only provides new theoretical support for the academic community but also offers valuable technical tools and development potential for the livestock industry.

In the aforementioned issue, occlusion remains a challenging problem in pose estimation due to the lack of visual information for key points. However, the human visual system can learn by observing the structural diversity of objects, and even in cases of severe occlusion, we can infer reasonable postures while rejecting illogical ones. Therefore, this study aims to develop a corresponding model capable of observing and learning sheep postures to achieve accurate recognition. Through long-term manual observation, common sheep postures include standing, lying, side lying, and standing–lying transitions. Based on these daily postures, we have developed a sheep posture estimation algorithm for real-world farm environments and experimentally validated the effectiveness of the proposed model. The study focuses on the classification of three primary daily postures (standing, lying, and side lying) and employs an image-based object detection approach to identify and classify the different postures within images. This method is expected to support the intelligent management of animal husbandry.

In summary, the main contributions of this study are as follows:

In practical farm environments, the high complexity of backgrounds and significant variations in image quality between daytime and nighttime conditions pose substantial challenges for sheep pose recognition. To address these issues, this study proposes an enhanced algorithm based on YOLOv8. The proposed method integrates a bidirectional feature pyramid network (BiFPN) for weighted feature fusion and a convolutional block attention module (CBAM) to improve detection accuracy. Additionally, the detection head is optimized to reduce both model parameters and computational overhead, thereby achieving efficient real-time performance.

This study designed and constructed a dataset comprising 1476 real-scene images captured at different time periods with varying degrees of occlusion. To enhance the diversity and robustness of the dataset, a series of data augmentation operations were applied, including flipping, cropping, noise addition, and other transformations.

Experimental results demonstrate that the improved YOLO v8 model (hereinafter referred to as YOLO v8-BCD) exhibits significant advantages in the trade-off between speed and accuracy on the sheep dataset. Its detection speed reaches 389.12 FPS, with a computational cost of 5.5 GFLOPs and a model size of 2.433 M parameters, achieving a detection accuracy of 91.7%.

## 2. Materials and Methods

### 2.1. Animal Pose Estimation

Pose estimation techniques have been widely applied in human sample analysis [28]. However, research on animal pose analysis is relatively limited, and most studies are based on the direct application of human pose estimation methods. In the field of animal pose estimation, researchers typically adopt two main estimation approaches: bottom-up and top-down methods. The bottom-up method first identifies local features in an image, such as key points [29,30], and then uses these local features to infer the overall pose. This approach is like starting with the smallest units of the image and progressively constructing an understanding of the entire pose. Its advantage lies in its ability to stably detect key points even in complex scenarios, such as environments with multiple animals interacting. However, the bottom-up approach requires high-resolution input images to better capture targets at different scales, which may involve a trade-off between processing speed and accuracy.

On the other hand, the top-down method adopts a different strategy. It first uses an object detector to identify and locate the individual animals in the image, then performs pose estimation analysis within predefined regions. The key advantage of this approach is that, by pre-identifying regions of interest, it reduces the amount of data that needs to be processed, thereby improving processing speed while maintaining high recognition accuracy. This method is particularly suitable for situations with fewer animals in the image, as it allows the use of lower resolution input images, effectively reducing computational load. Additionally, the top-down method can provide more precise key point localization by conducting fine-grained analysis within the local region, especially when the animal’s posture does not change significantly, or the background is relatively simple.

### 2.2. Dataset Construction

**Figure 1:**
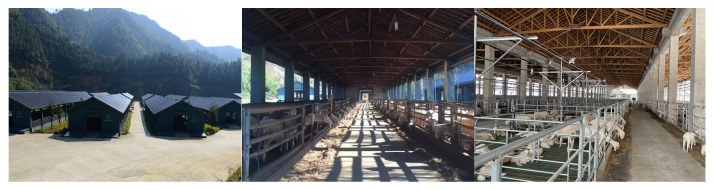
Sheep breeding site.

**Figure 2:**
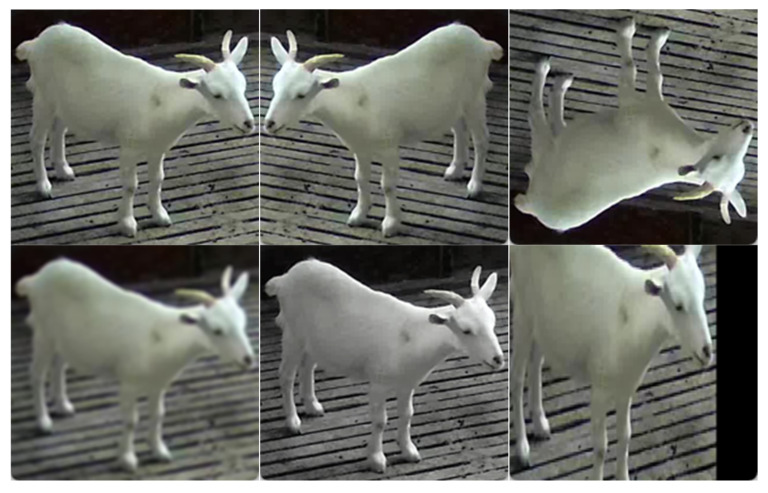
Data augmentation operations.

The sheep dataset used in this study was provided by Zhejiang Saino Ecological Agriculture Co., Ltd., located in Yiyi Village, Yiqian Town, Lin’an District, Hangzhou, Zhejiang Province. The farm covers an area of over 15,000 square meters and currently houses more than 12,000 sheep. Figure 1 illustrates the overall layout of the farm. The experiment was conducted in a separate shed, with the camera installed at the entrance of the sheep pen to observe and record the sheep’s behavior from this vantage point. The data collection period spanned from 15 June 2024, to 15 August 2024. A single RGB camera with a resolution of 2560 × 1440 pixels was used for filming. After data collection, clear and representative frames were manually selected from the recorded videos. Based on the original 1476 images, a series of data augmentation operations—including flipping, cropping, noise addition, and other transformations—were applied to enhance the diversity and robustness of the dataset. Figure 2 demonstrates various data augmentation operations employed in this study. These operations aim to simulate real-world variations in illumination, occlusion, and spatial configurations, thereby improving the generalization capability of the model during training. The LabelMe annotation tool was then used to label the positions and behaviors of the sheep. The dataset was divided into training, validation, and test sets in the following manner: 10% of the data was set aside as the test set, while the remaining 90% was split into an 80% training set and a 20% validation set.

### 2.3. Detection Network

In this study, we employ the YOLO (You Only Look Once) network for real-time object detection. YOLO, proposed by Joseph Redmon and colleagues in 2016 [31,32], is a popular single-stage detection algorithm. Unlike traditional two-stage detection algorithms, such as Faster R-CNN, YOLO simplifies object detection into a single regression problem. By leveraging deep convolutional neural networks, YOLO directly predicts object bounding boxes and class probabilities from image pixels, significantly enhancing detection speed while maintaining high accuracy, making it capable of real-time processing.

**Figure 3:**
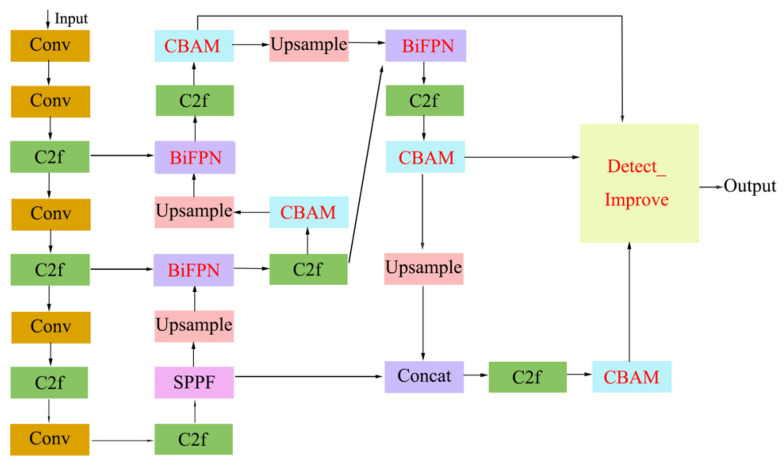
YOLO v8-BCD network structure.

For this study, we have made improvements to the YOLO v8 network to better accommodate the needs of sheep posture detection. The specific improvements include the introduction of the BiFPN weighted feature fusion network and attention mechanism to enhance the model’s feature focusing capability, as well as the optimization of the detection head to further improve detection accuracy while reducing parameter size and computational cost. Figure 3 illustrates the architecture of the modified YOLOv8 network, with the enhanced modules are highlighted in red text. The specific functionalities of these modules will be elaborated in subsequent sections.

**Figure 4:**
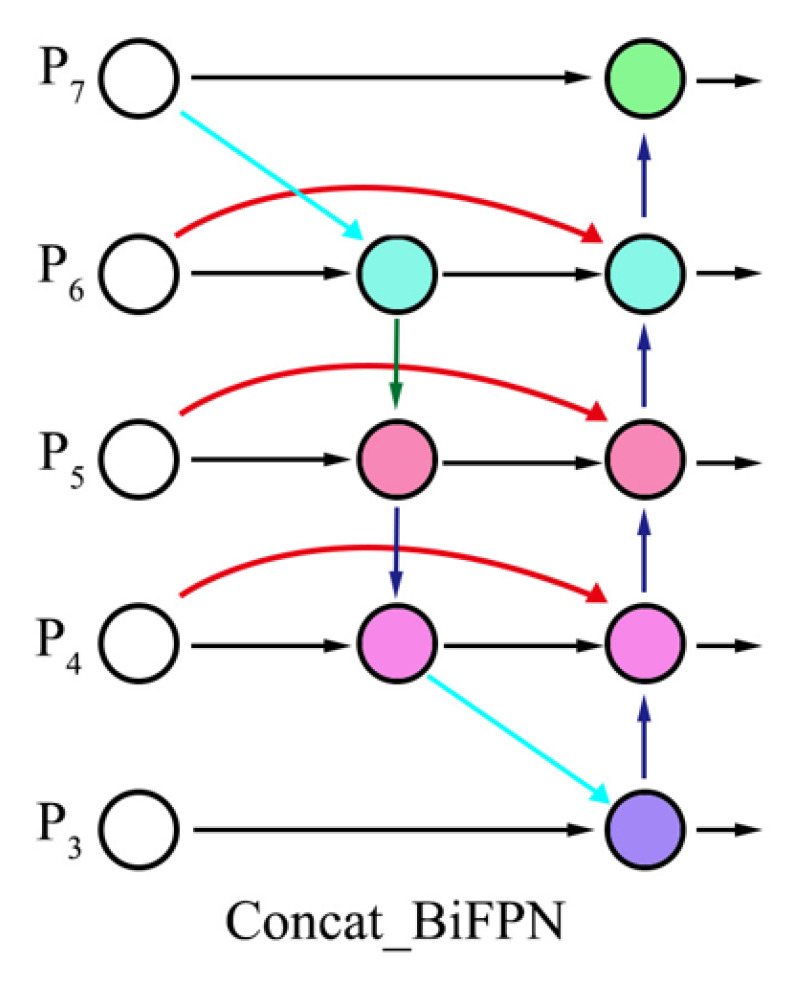
BiFPN module.

BiFPN: The BiFPN is primarily composed of multiple bidirectional feature fusion layers. In each layer, it not only integrates feature maps from different scales but also introduces bidirectional information flow, including a top-down and a bottom-up pathway. In the bottom-up pathway, low-resolution feature maps are integrated with high-resolution counterparts through up-sampling operations, enabling the propagation of high-level semantic information to lower layers and thereby enhancing the representational capacity of low-level features. Conversely, the top-down pathway incorporates down-sampling to transfer detailed spatial information from lower layers to higher levels, effectively compensating for the loss of fine-grained details in high-level feature representations. This bidirectional information flow mechanism allows for comprehensive interaction between features at different scales, resulting in more representative fused features. Additionally, BiFPN employs a learnable weight allocation strategy. During the feature fusion process, a learnable weight is assigned to each input feature map, enabling the network to automatically adjust the importance of different feature maps based on the characteristics of the data, further improving the fusion effectiveness. Figure 4 illustrates the structure of the BiFPN module.

Given that input features at different resolutions typically contribute unequally to output features, BiFPN addresses this issue by incorporating learnable weights for each input, enabling the network to adaptively assign appropriate importance levels to distinct feature maps. To ensure that each $w_{i}$ value is non-negative ($w_{i}$ ≥ 0), a rectified linear unit activation function is applied after each $w_{i}$. The final BiFPN integrates bidirectional cross-scale connections and fast normalized fusion. As a specific example, the two fused features in the fourth layer are described by Equations (1) and (2). In the formulation, $P_{4}^{\mathrm{td}}$ denotes the intermediate feature within the top-down pathway at Level 4. $w_{i}$ represents the learnable weight parameter, while $P_{4}^{in}$ corresponds to the input feature of Level 4. * represents convolution operations. $Resize(P_{5}^{in})$ indicates the resized feature derived from Level 5’s input through up-sampling or down-sampling to match Level 4’s resolution. ∈ is a minimal constant (e.g., 0.0001) introduced to prevent division by zero and ensure numerical stability. The final output feature of Level 4 is designated as $P_{4}^{out}$.

(1) $$P_{4}^{\mathrm{td}}=Block(\frac{w_{1}*P_{4}^{in}+w_{2}*Resize(P_{5}^{in})}{w_{1}+w_{2}+\in })$$

(2) $$P_{4}^{out}=Block(\frac{{w^{\prime }}_{1}*P_{4}^{in}+{w^{\prime }}_{2}*P_{4}^{td}+{w^{\prime }}_{3}*Resize(P_{3}^{out})}{{w^{\prime }}_{1}+{w^{\prime }}_{2}+{w^{\prime }}_{3}+\in })$$

**Figure 5:**
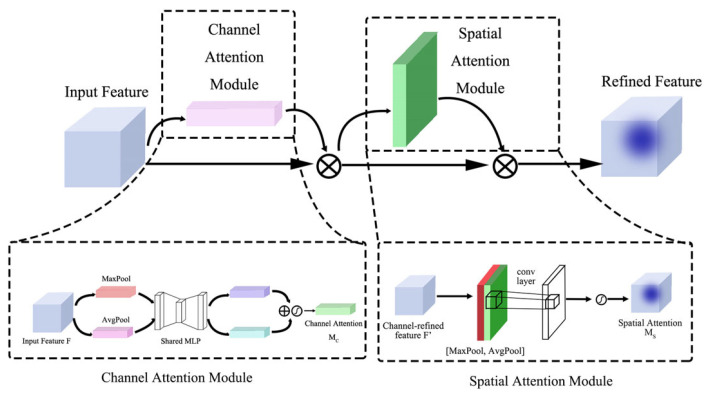
CBAM attention mechanism.

Attention mechanism: The attention mechanism mimics the attention principles in the human visual system by assigning differential weights to different pieces of information in the input data. This allows the model to focus on the most important parts of the input, thereby enhancing its performance and generalization ability. Specifically, in this study, we adopt the convolutional block attention module (CBAM [33]), which takes intermediate feature maps $F\in R^{C\times H\times W}$ as input and sequentially derives a one-dimensional channel attention map $M_{C}\in R^{C\times 1\times 1}$ and a two-dimensional spatial attention map $M_{S}\in R^{1\times H\times W}$, as illustrated in Figure 5. The CBAM module first applies the channel attention module to weight the feature responses of different channels, enhancing the channels that contain more semantic information in the feature map. Then, the spatial attention module further focuses on important spatial locations within the feature map, highlighting regions with high significance. The design of CBAM allows it to function as a plug-and-play module, easily integrated into existing convolutional neural network (CNN) architectures and trained end-to-end alongside the base network. By applying the attention mechanism sequentially in both the channel and spatial dimensions, CBAM effectively enhances the model’s ability to perceive key features, improving performance across various vision tasks. The overall attention process can be summarized as follows:

(3) $$M_{C}(F)=\sigma (MLP(AvgPool(F))+MLP(MaxPool(F)))$$

(4) $$F^{\prime }=Mc(F)⊗F$$

(5) $$Ms=\sigma (f^{7\times 7}([AvgPool(F^{\prime });MaxPool(F^{\prime })]))$$

(6) $$F^{\prime \prime }=Ms(F^{\prime })⊗F^{\prime }$$

the symbol ⊗ denotes element-wise multiplication.

### 2.4. Key Improvements

**Figure 6:**
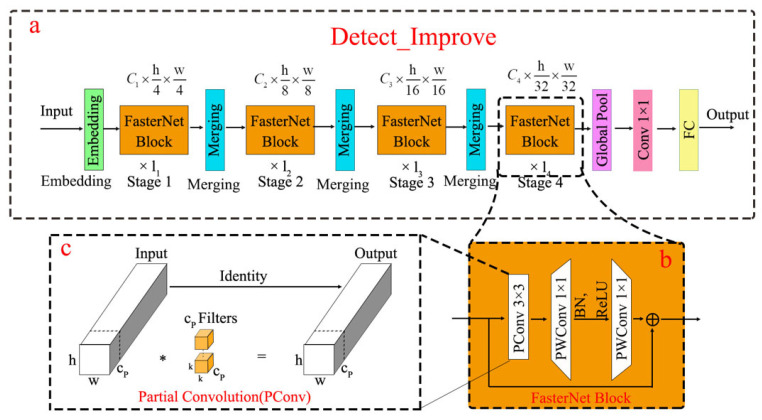
Improved framework for detection head. * represents convolution operations.

Detection head improvement: This study optimizes the detection head of YOLO v8 to enhance both efficiency and accuracy in object detection. As the core component of object detection models, the detection head is responsible for predicting bounding boxes and class probabilities from feature maps. To improve the model’s adaptability on computationally constrained devices, we introduce partial convolution (PConv, shown in Figure 6c) and an enhanced FasterNet block structure (illustrated in Figure 6b) into the detection head. PConv serves as a simple yet effective operator that simultaneously reduces computational redundancy and memory access. Compared with standard convolution, it achieves lower FLOPs while maintaining higher FLOPs than depth-wise separable convolution (DWConv), thereby better leveraging device computational capabilities.

The FLOPs of standard convolution are calculated as shown in Equation (7), where h and w represent the height and width of the feature map, k denotes the kernel size, and c_p_ is the number of channels involved in the convolution computation. For PConv, its FLOPs are formulated in Equation (8). When computing only a subset of channels (with the partial ratio denoted as r, as shown in Equation (9)). PConv achieves a FLOPs of only 1/16th that of standard Conv (when the partial ratio r = 0.25), thereby demonstrating its exceptional efficiency in computational cost reduction.

(7) $$FLOPs_{Conv}=h\times w\times k^{2}\times c^{2}$$

(8) $$FLOPs_{PConv}=h\times w\times k^{2}\times c_{p}^{2}=\frac{FLOPs_{Conv}}{16}$$

(9) $$r=\frac{c_{p}}{c}=0.25$$

Memory access of standard Conv: For standard convolution with equal input and output feature map channels, where the feature map dimensions are h × w × c and the kernel size is k^2^ × c^2^, the memory access (MAC) is formulated in Equation (10). Since the kernel size is typically much smaller than the feature map dimensions (as shown in Equation (11)), the MAC can be simplified to Equation (12).

(10) $$Memory_{Conv}\approx h\times w\times 2c+k^{2}\times c^{2}$$

(11) $$h\times w\times 2c\ll k^{2}\times c^{2}$$

(12) $$Memory_{Conv}\approx h\times w\times 2c$$

Memory access of PConv: Under identical channel configurations, the MAC of PConv can be simplified to Equation (13). When the partial ratio r = 0.25, PConv achieves a MAC of 1/4th that of standard Conv, demonstrating its significant advantage in reducing memory access—a critical factor for enhancing computational efficiency and reducing latency, particularly in real-time or resource-limited applications.

(13) $$Memory_{PConv}\approx h\times w\times 2c_{p}=h\times w\times \frac{c}{2}=\frac{Memory_{Conv}}{4}$$

PConv performs convolution on only a subset of input channels, reducing both computational load and memory access. To fully utilize information across all channels, a pointwise convolution (PWConv, 1 × 1 convolution) is appended after PConv. PWConv propagates the features extracted by PConv to all channels. This combination ensures that the overall computational efficiency of PConv and PWConv remains superior. The effective receptive field of the PConv + PWConv pair on the input feature map resembles a “T-shaped convolution” (T-shaped Conv), which prioritizes the central regions of the kernel rather than uniformly processing the entire kernel area, as in standard convolution. The FLOPs of T-shaped Conv are formulated in Equation (14), while the combined FLOPs of PConv and PWConv are derived in Equation (15). This architecture not only reduces computational costs but also preserves robust feature extraction capabilities.

(14) $$FLOPs_{PWConv}=h\times w\times c_{p}\times c$$

(15) $$FLOPs_{Total}=\frac{FLOPs_{Conv}}{16}+\frac{FLOPs_{Conv}}{4}=\frac{5}{16}\times FLOPs_{Conv}$$

The FasterNet architecture is designed around PConv and PWConv. It comprises four hierarchical stages. Prior to each stage, an embedding layer (employing a 4 × 4 convolution with stride 4 for initial feature extraction) or a merging layer (using a 2 × 2 convolution with stride 2 for down-sampling and channel expansion between stages) is applied to perform spatial down-sampling and channel scaling. Each FasterNet block contains a PConv layer followed by two PWConv layers. This design resembles an inverted residual block, where the intermediate layer expands the channel count and reuses input features via a shortcut connection. To preserve feature diversity while minimizing latency, normalization and activation layers are applied only after the intermediate PWConv layer within each block. The final three layers of FasterNet include global average pooling, a 1 × 1 convolution, and a fully connected layer for feature transformation and classification. Our enhanced version of FasterNet maintains computational efficiency while significantly improving object detection accuracy and robustness, particularly excelling in handling occlusions and multi-scale targets. The introduced techniques effectively reduce model parameters and computational complexity while enhancing detection precision. The computational workflow of the detection head is illustrated in Figure 6a, The Faster block framework is illustrated in Figure 6b, and the PConv framework is illustrated in Figure 6c.

## 3. Results

### 3.1. Experimental Settings

In this study, both the training and testing of the model were conducted on a workstation equipped with an Intel Core i5-14600KF 3. 5 GHz CPU (The manufacturer of the device is Intel Corporation, headquartered in Santa Clara, CA, USA), 32 GB of RAM, and running the Windows 10 (64-bit) operating system. The system is supported by an NVIDIA GeForce RTX 4060 Ti GPU with 8 GB of VRAM (The manufacturer of the device is NVIDIA, headquartered in Santa Clara, CA, USA), providing hardware acceleration. The programming environment used Python version 3. 8, with PyTorch 2.3.0 as the deep learning framework. To optimize the training process, we employed the Adam optimizer with a batch size of 16. The initial learning rate was set to 0.001, chosen to balance the convergence speed and learning efficiency while preventing instability in model training that could result from an excessively high learning rate. Additionally, to accelerate learning and avoid local minima, we incorporated a momentum decay strategy with a decay factor of 0.93. Furthermore, to improve the model’s generalization ability, we introduced a weight decay mechanism with a coefficient of 0.0005 to reduce the risk of overfitting.

### 3.2. Experimental Details and Evaluation Metrics

In the experimental section, the improved YOLO v8 model proposed in this paper is compared with traditional detection networks, including Faster R-CNN, SSD, DETR, and YOLO v5. Through comparisons with these models, this paper evaluates detection accuracy, model size, and computational cost. Precision and recall, commonly used evaluation metrics in object detection, are calculated as shown in Equations (16) and (17).

(16) $$precision=\frac{TP}{TP+FP}$$

(17) $$recall=\frac{TP}{TP+FN}$$

TP refers to the samples correctly identified as positive, TN to those correctly identified as negative, FP to negative samples incorrectly classified as positive, and FN to positive samples missed by the model. To comprehensively evaluate both precision and recall, this study adopts average precision (AP) as a performance metric, with its calculation method shown in Equation (18) in the referenced literature. Additionally, to assess the overall performance of the model in multi-class detection tasks, we calculate the mean average precision (mAP) across all categories, as shown in Equation (19), where the letter k denotes the total number of classes. Through the values of AP and mAP, we can more accurately evaluate the model’s performance in object detection tasks.

(18) $$AP=\int_{0}^{1}P(r)dr$$

(19) $$mAP=\frac{\sum_{i=1}^{K}AP_{i}}{k}$$

### 3.3. Comprehensive Comparison of Different Models

**Figure 7:**
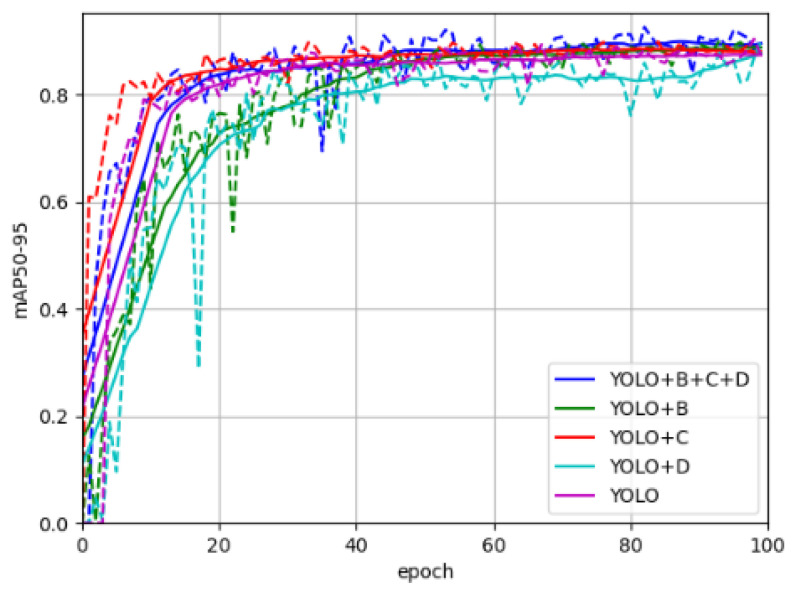
The results of mAP50.

Figure 7 presents the comparison results of different optimized models in terms of mAP50 during the first 100 training iterations. All models exhibited a progressive improvement in mAP50 as training epochs increased, eventually reaching performance plateaus. This convergence pattern validates the effectiveness of the training process by demonstrating consistent performance stabilization across models. The model incorporating three stacked modules achieved the highest final mAP50 value, indicating that the synergistic combination of these modules significantly enhances model performance. These experimental results substantiate the efficacy of the proposed improvements.

In the experimental analysis of eight object detection models, including Faster R-CNN, SSD, DETR, YOLO v5, YOLO v8, YOLO v10, YOLO v11, and YOLO v8-BCD, a comprehensive evaluation was conducted across multiple dimensions, including precision, recall, detection accuracy for different postures, mAP50, model parameter size, and computational complexity.

Table 1 presents the recognition performance metrics and computational complexity of various detection models for different postures (standing, lying, and side lying). The optimal bolded display for each item. By evaluating different metrics, the performance of each model is thoroughly explored. Precision reflects the model’s ability to accurately identify targets, effectively reducing false positives. Recall represents the proportion of correctly identified positive samples among all actual positive samples, with higher recall indicating the model’s effectiveness in locating targets. The evaluation of the three postures demonstrates the strong detection capabilities of the models across different postures. Mean average precision serves as a critical indicator for measuring the overall performance of a model. The Faster R-CNN model performs well in recall, standing posture, lying posture, and mAP50. The YOLO v8-BCD model, however, achieves the best results in precision, side-lying posture, parameter size, and computational cost. Specifically, the parameter size and floating-point operations of YOLO v8-BCD are 2.433 M and 5.5 G, respectively, the lowest among all models. Even the newly published YOLO v10 and YOLO v11 are not as effective as YOLO v8-BCD. This demonstrates that the YOLO v8-BCD model is more streamlined in structural design, enabling efficient operation on devices with limited computational resources, significantly reducing hardware requirements and operational costs. Although the YOLO v8-BCD model is not the best in every metric, it stands out in overall performance. It achieves an excellent balance between efficiency and accuracy, making it the preferred model for sheep behavior detection tasks.

### 3.4. Comparison Under Different Lighting Conditions

To investigate the enhanced performance of the YOLO v8-BCD model under varying illumination conditions, we partitioned the dataset into two subsets: daytime and nighttime. Comparative experiments were conducted on both the baseline YOLO v8 and the proposed YOLO v8-BCD models. The daytime results are summarized in Table 2, while the nighttime results are presented in Table 3.

As evident from the two tables, the YOLO v8-BCD model demonstrates superior performance across nearly all metrics. Daytime results generally outperform nighttime results, which can be attributed to better illumination conditions and higher image clarity in daytime scenarios. Notably, on nighttime images, the YOLO v8-BCD achieves an mAP50 of 89.9, significantly surpassing the baseline YOLO v8’s mAP50 of 88.5. These improvements validate that the proposed enhancements effectively enhance the YOLO v8 framework, particularly in challenging low-light environments.

### 3.5. Ablation Study

To further validate the effectiveness of these improvement strategies, we conducted comprehensive ablation experiments. In these experiments, we evaluated the impact of adding the BiFPN weighted fusion network, the CBAM attention mechanism, and the Detect_Improve strategy on the performance of the YOLO v8 model individually. The optimal bolded display for each item. Additionally, we observed the performance of the model under different combinations of these strategies. The experimental results are shown in Table 4.

The experimental results demonstrate that the introduction of BiFPN significantly improves the model’s precision but leads to a reduction in recall. The addition of the CBAM attention mechanism, on the other hand, shows remarkable performance in enhancing mAP50. The implementation of the Detect_Improve strategy is particularly effective in improving precision, while also significantly increasing FPS by reducing model parameters and computational costs. Further analysis reveals that when BiFPN, CBAM, and Detect_Improve strategies are applied together, the model achieves an F1 score of 86.27%, the best result among all tested combinations. This indicates that the combination of these three strategies strikes a good balance between precision and recall. Additionally, the introduction of these strategies results in a significant improvement in mAP50, further validating their effectiveness. The ablation study results in this research show that BiFPN, CBAM, and Detect_Improve strategies can effectively enhance the detection performance of the YOLO v8 model. However, a trade-off must be made between precision, processing speed, and computational resources. Future work could explore the further optimization of these strategies to maintain high precision while minimizing the impact on real-time processing capability and computational resource requirements.

### 3.6. Heatmap Visualization Analysis

**Figure 8:**
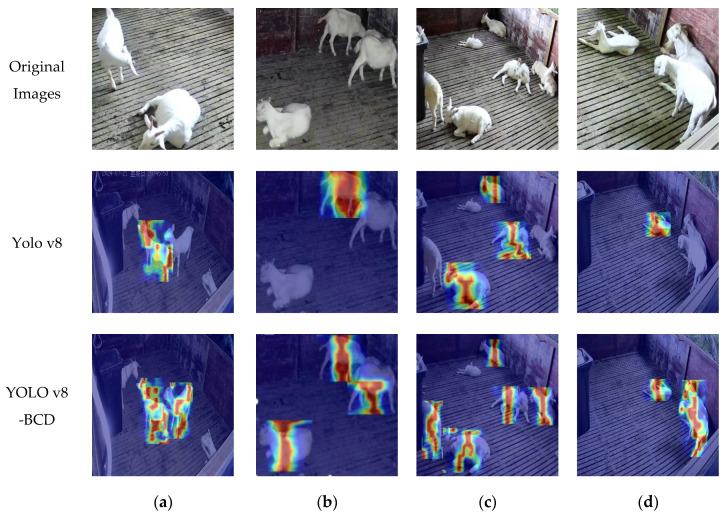
Comparison of heat maps before and after model optimization. The darker the color, the larger the data value, and the lighter the color, the smaller the data value. (**a**–**d**) are images of different poses and poses, respectively.

To better evaluate model adaptability from a visual perspective, we compare the heatmaps generated by YOLOv8 and YOLO v8-BCD, as illustrated in Figure 8, which highlights advancements in feature detection and localization precision. Heatmaps serve as an effective visualization tool, employing gradient color transitions to represent detection confidence, where cooler colors (such as blue or green) denote lower values and warmer colors (such as red or yellow) indicate higher values. As shown, compared to standard YOLOv8, the YOLO v8-BCD model demonstrates finer coverage and distribution of detection regions, with heightened intensity and clarity in regions of interest (ROIs). Notably, the YOLO v8-BCD model successfully identifies objects even in partially occluded areas. These enhancements indicate strengthened feature extraction capabilities and improved robustness in the detection process, resulting in outputs that align more precisely with the actual scenes depicted in the original images. Such advancements are particularly valuable for high-precision applications like livestock monitoring, where accurate object localization and segmentation are critical to operational success.

## 4. Discussion

The proposed YOLO v8-BCD model demonstrates significant performance improvements and practical utility in sheep pose recognition tasks. By integrating a BiFPN-based weighted feature fusion network, CBAM attention mechanism, and Detect_Improve strategy, the model achieves a balance between high precision and computational efficiency in complex farming scenarios. The BiFPN enhances multi-scale target perception through bidirectional cross-scale feature fusion. Combined with the CBAM mechanism, which focuses on critical features across channel and spatial dimensions, the model mitigates recall rate degradation while further elevating the mAP50 to 91.7%. The Detect_Improve strategy optimizes the detection head architecture, compressing parameters to 2.433 M, reducing computational costs to 5.5 GFLOPs, and achieving an inference speed of 389 FPS—demonstrating that the lightweight design ensures real-time performance without compromising accuracy.

**Figure 9:**
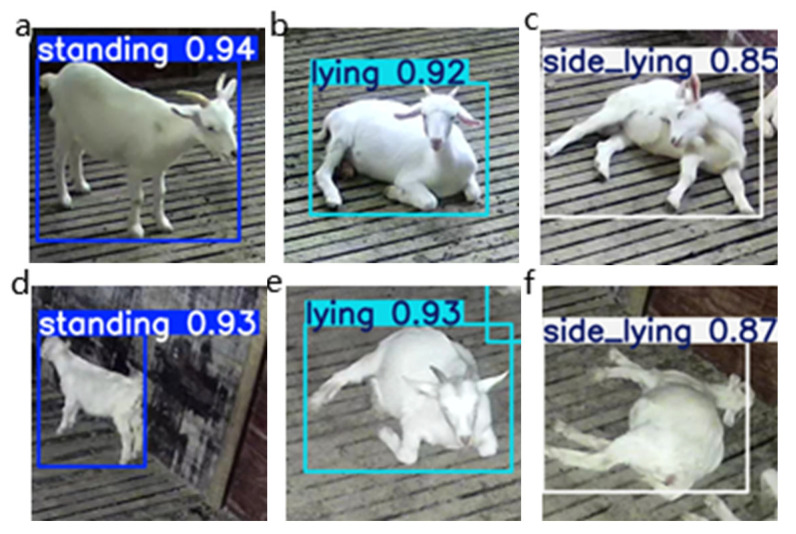
The actual performance of sheep during the day and at night when illuminated by cameras: (**a**–**c**) are daytime images and (**d**–**f**) are nighttime images.

The model’s adaptability is validated under diverse lighting conditions, as illustrated in Figure 9, which compares daytime performance with nighttime scenarios illuminated by camera-provided lighting. Figure 9a–c depict daytime images, while Figure 9d–f represent nighttime conditions. Despite noticeable declines in nighttime image quality (e.g., increased blur due to insufficient ambient light), the YOLO v8-BCD model maintains robust detection accuracy, underscoring its resilience to low-light challenges. Future work will focus on further optimizing the model for extreme environmental conditions.

## 5. Conclusions

The proposed YOLO v8-BCD model achieves a balance between precision and efficiency in sheep pose recognition tasks, offering a viable technical solution for intelligent livestock management. By integrating the BiFPN-weighted feature fusion network, CBAM attention mechanism, and Detect_Improve optimization strategy, the model demonstrates superior performance in complex scenarios: detection accuracy improves to 91.7% mAP50, with a 19% reduction in parameters (2.433 M) and a 32% decrease in computational load (5.5 GFLOPs), while achieving real-time inference at 389 FPS. Additionally, the model exhibits robust environmental adaptability, maintaining high recognition accuracy (89.9% mAP50) under varying illumination conditions, particularly in low-light nighttime environments with blurred images, as detailed in Table 3.

Our research will focus on three key directions: (1) further optimizing the YOLO v8-BCD algorithm to address more complex farming environments and stricter real-time requirements; (2) curating higher-quality datasets tailored to practical agricultural needs; and (3) extending static image-based pose estimation to video-based temporal analysis by integrating sequential information, thereby enhancing real-time performance and accuracy for dynamic monitoring in livestock management. We anticipate that these advancements will significantly enhance intelligent livestock management systems and broaden the model’s applicability to diverse agricultural and industrial domains.

## Figures and Tables

**Table 1:** Comparative experimental results of different models.

Model	Precision	Recall	Standing	Lying	Side Lying	mAP50	Params (M)	GFLOPs (G)
Faster RCNN	74.52	**92.49**	**97.82**	**88.14**	89.51	**91.82**	28.480	941.169
SSD	82.76	81.29	96.49	86.13	82.22	88.28	26.285	62.747
DETR	73.25	91.74	97.18	85.12	85.05	89.12	36.762	114.249
YOLO v5	87.21	82.68	95.30	85.30	85.70	88.80	2.504	7.1
YOLO v8	85.23	85.42	94.30	84.30	86.70	88.50	3.006	8.1
YOLO v10	81.73	83.22	94.60	86.20	86.30	89.00	2.696	8.2
YOLO v11	87.91	77.83	94.00	86.30	88.00	89.40	2.583	6.3
YOLO v8-BCD	**91.84**	81.33	96.90	86.40	**91.90**	91.70	**2.433**	**5.5**

**Table 2:** The results of the daytime dataset.

Model	Precision	Recall	Standing	Lying	Side Lying	mAP50
YOLO v8	84.61	83.83	95.20	96.10	82.20	91.20
YOLO v8-BCD	86.22	86.56	96.50	93.70	87.90	92.70

**Table 3:** The results of the nighttime dataset.

Model	Precision	Recall	Standing	Lying	Side Lying	mAP50
YOLO v8	83.84	78.51	88.60	84.40	89.80	87.60
YOLO v8-BCD	84.63	82.61	95.80	85.80	88.20	89.90

**Table 4:** Results of ablation experiment.

Model	BiFPN	CBAM	Detect_ Improve	Precision	Recall	F1	mAP50	FPS (ms)	Params (M)	GFLOPs (G)
YOLO v8	no	no	no	85.23	**85.42**	85.32	88.5%	312.33	3.006	8.1
YOLO v8	add	no	no	88.74	79.75	84.01	91.1%	265.78	3.006	8.1
YOLO v8	no	add	no	86.33	84.60	85.46	91.5%	327.45	3.019	8.1
YOLO v8	no	no	add	91.20	73.78	81.57	88.2%	378.21	**2.420**	**5.5**
YOLO v8	add	add	no	87.05	81.32	84.09	**92.6%**	342.96	3.019	8.1
YOLO v8	add	no	add	88.46	81.12	84.63	90.9%	356.89	**2.420**	**5.5**
YOLO v8	no	add	add	**93.07**	69.13	79.33	91.2%	**395.67**	2.433	**5.5**
YOLO v8	add	add	add	91.84	81.33	**86.27**	91.7%	389.12	2.433	**5.5**

## Data Availability

The data are not publicly available due to ongoing study.
